# The Impact of Medical Student Participation in Emergency Medicine Patient Care on Departmental Press Ganey Scores

**DOI:** 10.5811/westjem.2015.9.27321

**Published:** 2015-10-22

**Authors:** Aaron W. Bernard, Daniel R. Martin, Mark G. Moseley, Nicholas E. Kman, Sorabh Khandelwal, Daniel Carpenter, David P. Way, Jeffrey M. Caterino

**Affiliations:** * Quinnipiac University, Frank H. Netter MD School of Medicine, Hamden, Connecticut; † Ohio State University College of Medicine, Department of Emergency Medicine, Columbus, Ohio; ‡ Ohio State University, Department of Biomedical Informatics, Columbus, Ohio

## Abstract

**Introduction:**

Press Ganey (PG) scores are used by public entities to gauge the quality of patient care from medical facilities in the United States. Academic health centers (AHCs) are charged with educating the new generation of doctors, but rely heavily on PG scores for their business operation. AHCs need to know what impact medical student involvement has on patient care and their PG scores.

**Purpose:**

We sought to identify the impact students have on emergency department (ED) PG scores related to overall visit and the treating physician’s performance.

**Methods:**

This was a retrospective, observational cohort study of discharged ED patients who completed PG satisfaction surveys at one academic, and one community-based ED. Outcomes were responses to questions about the overall visit assessment and doctor’s care, measured on a five-point scale. We compared the distribution of responses for each question through proportions with 95% confidence intervals (CIs) stratified by medical student participation. For each question, we constructed a multivariable ordinal logistic regression model including medical student involvement and other independent variables known to affect PG scores.

**Results:**

We analyzed 2,753 encounters, of which 259 (9.4%) had medical student involvement. For all questions, there were no appreciable differences in patient responses when stratifying by medical student involvement. In regression models, medical student involvement was not associated with PG score for any outcome, including overall rating of care (odds ratio [OR] 1.10, 95% CI [0.90–1.34]) or likelihood of recommending our EDs (OR 1.07, 95% CI [0.86–1.32]). Findings were similar when each ED was analyzed individually.

**Conclusion:**

We found that medical student involvement in patient care did not adversely impact ED PG scores in discharged patients. Neither overall scores nor physician-specific scores were impacted. Results were similar at both the academic medical center and the community teaching hospital at our institution.

## INTRODUCTION

Press Ganey (PG) scores are an important marker of quality medical care, used by hospital administrators, healthcare consumers, and payers.^1^–^3^ Subsequently, reimbursements are being linked to these measurements.^4^ Researchers have identified variables associated with PG scores including interpersonal interactions, patient communication, and perceived wait time.^1^–^4^ Other factors such as age, race/ethnicity, triage acuity, and arrival time have also been suggested to affect PG scores.^1^–^5^

The impact of medical student involvement on an emergency departments (ED’s) PG scores is not well defined in the literature. A convenience sample of 145 patients in an ED located in Ireland suggested positive patient attitudes towards medical students.^6^ Similar studies of non-ED ambulatory settings have also reported positive patient opinions about medical students.^7^–^9^

ED student rotations have been increasing over the last decade.^10^,^11^ Medical school enrollment is also increasing, requiring additional clinical teaching sites to accommodate demand.^12^ While reports from other settings provide reassurance that medical students are well received by patients, ED studies are limited.^6^–^9^ Furthermore, recent literature indicates that patients have difficulty distinguishing between various providers.^13^,^14^ Accordingly, there is a possibility that students could not only impact an ED’s overall PG scores, but also the scores of physician providers. The potential for negative student impacts on PG scores could hinder developing partnerships between EDs and medical schools, and could ultimately harm provider reimbursement. Therefore, a better understanding of the relationship between medical students and EDs’ PG measures is needed. The goal of this investigation was to determine whether medical student involvement in emergency care impacts our ED’s PG scores.

## METHODS

### Study Design and Setting

We conducted a retrospective, observational cohort study examining the relationship between medical student involvement in ED care and PG survey scores, a common surrogate measure of patient satisfaction. The study was approved by the hospital institutional review board and was conducted in compliance with the Strengthening the Reporting of Observational studies in Epidemiology statement.^15^

The Ohio State University medical facility provides patient care in two EDs. The first is an academic, tertiary care, Level I trauma center with a volume of 72,000 patient visits per year (the “academic ED”). The academic ED patient population is diverse with regard to ethnicity/race and economic status. The second is a community teaching ED with a volume of 50,000 patient visits per year (the “community ED”). The community ED patient population is primarily African-American and of lower economic status. Both sites are staffed by the same physician group. Staffing consists of board-certified emergency physicians, emergency medicine resident physicians, and rotating resident physicians. The academic ED averages seven residents per shift and the majority of patients have a resident involved in their care. Advanced-Practice providers (APPs; nurse practitioners and physician assistants) primarily care for lower acuity patients. The community ED typically has either one resident or one APP on each shift, with additional APP coverage for lower acuity patients.

The Ohio State University College of Medicine enrolls 210 students per year. All fourth-year medical students take a required, four-week ED clerkship at one of 10 EDs in the Central Ohio area. About 95, (45%) are assigned to the two study EDs, averaging about eight per month. Additionally, the study EDs take two third-year elective students per month.

Medical students select patients who have been identified in the electronic medical record (EMR) as having been triaged and placed in a room, i.e. “waiting for provider.” Once a patient is selected, the student performs a focused history and physical examination, and then presents the case to the supervising physician. Students are encouraged to select patients with a broad range of chief complaints and triage acuity. Critically ill patients are rarely labeled as “waiting for provider,” and therefore students are not involved with their care. Students are not provided any specific education about PG surveys or patient satisfaction.

### Selection of Participants

All patients seen at either the Ohio State University Main Hospital ED or their affiliate community hospital ED and who completed a PG survey for visits between December 1, 2011, and December 31, 2012, were eligible for the study. Eligible patients receive a survey if they were age 18 or over and discharged from the ED. Patients admitted or placed in observation units were not eligible and were excluded from the study. Patients were also excluded if they failed to answer both primary outcome items: H.1 “Overall rating of care received during your visit” and H.2 “Likelihood of your recommending our ED to others.” Exclusion criteria also included lack of attending physician involvement in care or missing documentation about attending providers.

### Measurement

Adult patients discharged from the EDs are randomly selected by PG and receive a telephone survey call. Trained PG personnel administer surveys. Five call attempts are made at different times for each patient selected. Additional patients are called until a quota of 230 completed surveys per month for both EDs combined is reached. PG does not report traditional response rates for phone surveys, but they estimate a rate of one completed survey for every eight patients called.

The PG ED satisfaction survey consists of 31 questions organized into eight sections: arrival, nurses, doctors, tests, family or friends, personal/insurance information, personal issues, and overall assessment. PG reports rigorous psychometric testing as part of survey design and evaluation to provide reliable and valid data.^16^

Data for this study was electronically obtained from the institution’s central data repository. Clinical data is completed using the EPIC (EPIC, Verona, WI) EMR. Variables included patient and visit characteristics as well as results of the PG survey. We performed range checking for all variables.

The ED visited (academic or community ED) was identified. Abstracted patient characteristics included age and race/ethnicity, which was coded as White, Hispanic, Black, or other. Hispanics were combined with Whites for analyses because of the small number of Hispanic patients resulting in complete or quasi-complete separation in the multivariable ordinal models.

We categorized month of visit by season: winter (January–March), spring (April–June), summer (July–September), or fall (October–December). Arrival time was categorized by shift: day (7am–3pm), evening (3pm–11pm), or night (11pm–7am). Emergency severity index (ESI) at triage was calculated by nursing staff for all ED patients.^17^ Given a limited number of Level I encounters (highest acuity patients often by-pass the ED) and level 5 encounters, the ESI was coded as three levels: ESI 1–2, ESI 3, and ESI 4–5. Primary payer type was coded as managed care, private insurance, Medicaid, Medicare, other governmental payer, or self-pay. We identified ED length of stay as time of arrival until time of discharge. Because of outliers and non-linear distribution, length of stay variable was divided into quartiles. We created dichotomous variables to note the use of plain radiographs, computed tomography (CT), and laboratory tests for each visit.

Provider variables included whether a medical student or resident physician participated in the patient care, and the attending physician who discharged the patient. We excluded patients with missing responses to a specific PG question, from analysis of that question.

### Outcomes

Outcome variables were patient responses to PG questions related to overall satisfaction and to satisfaction with physician care. All responses were ordinal variables scored on a five-point scale with 1 being very poor and 5 being very good. The items of primary interest were overall rating of ED care (H.1) and likelihood of recommending ED to others (H.2). Of secondary interest were four items regarding physician behavior (C.1–C.4).

### Analysis

Descriptive data included proportions, means with standard deviations (SD), and medians with interquartile range (IQR) as appropriate. Normality was tested using the Shapiro-Wilk test. We made comparisons of patient responses between those who experienced medical student participation and those who did not using chi-square tests. Analyses were performed with STATA v12 (STATACorp, College Station, TX).

We first reported the distribution of patient responses to each of the study questions stratified by medical student participation in the patient’s care. We next constructed ordinal logistic regression models clustered by attending physician for each question. After constructing an initial unadjusted univariate model including only medical student involvement and the clustering, we then constructed adjusted multivariable models for each outcome. Independent variables were chosen a priori from factors previously shown to affect patient satisfaction scores.^1^–^5^ These included medical student involvement, ED visited, age, race, season of visit, time of arrival, ESI level, payer type, ED length of stay, ordering of ≥1 radiograph, ≥1 CT, ≥1 laboratory test, and resident involvement. The highest level of recommendation (5–very good) was used as the reference group. Resulting odds ratios (OR) >1 indicate greater odds of having lower satisfaction scores. In each model we tested for interactions between medical student involvement and: patient age, ESI level, and resident involvement.

Variables in each model were tested for violation of the proportional hazards (parallel lines) assumption using the Brant test. A partial proportional odds model was then created using the STATA gologit2 command to allow variables violating the assumption to vary across response levels.^18^,^19^ Variables not violating the proportional odds assumption continued to be held constant across response levels. To determine the best fitting model for each question, we calculated the Akaike Information Criterion (AIC) for each of the models created for that question.^20^ As a sensitivity analysis, we analyzed each question for each of the two EDs individually. We also performed a sensitivity analysis adding a satisfaction with nursing care variable to the model. This was based on the average score of each patient in the nursing-related questions of the PG survey.

Sample size requirements for multivariable ordinal logistic regression are not clearly defined.^21^ The rule of thumb for logistic regression is 10 outcome events per independent variable. In 13 months, we expected to have 2,990 surveys for analysis and we expected that 20% (n=598) of our subjects would report satisfaction scores of <5. This would provide 20 patients with scores <5 (i.e., 20 outcome events) for each of up to 30 independent variables (accounting for multilevel nature of several categorical variables).

Finally, because PG surveys are completed by a minority of the discharged ED population, we obtained descriptive statistics on the entire discharged ED population during the study time period to identify differences in characteristics between patients completing the survey and those who did not.

## RESULTS

**Figure 1:**
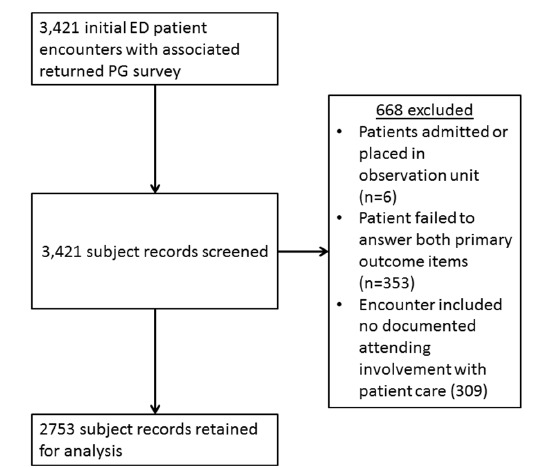
Flow chart describing identification of patient subjects for study. *ED,* emergency department; *PG,* press ganey

Initially, 3,421 ED encounters with returned PG surveys were identified. Exclusion criteria eliminated 668, leaving 2,753 surveys for the study: (353 were missing answers to the primary outcome questions, 295 lacked attending involvement, 14 were missing documentation of specific attending involved, and 6 were admitted/observation patients; see Figure 1 for a flow chart description). Missing outcome data included a lack of patient response to questions C.1–C.4: 52 missing for C1, 56 for C.2, 66 for C.3, and 67 for C.4. Forty-five patients did not respond to any of the four questions. We retained all patients for the analysis of the primary outcomes (questions H.1 and H.2), but those with missing responses to specific questions C.1–C.4 were excluded from analysis of that question. Although our initial expectations were to have 2,990 surveys for the study, for the primary analysis there were greater than 40 outcome events (score less than 5 or “very good”) per independent variable or variable level in each model. This is well above the recommended 10 outcome events per independent variable.^21^

Population demographics are shown in v 1. For the race/ethnicity variable, the White or Hispanic category included 52 Hispanics and the other category included 50 Asians and 5 Native Americans. There were 42 attending physicians who worked shifts during the study period (December 1, 2011 through December 31, 2012). The 42 physicians encountered an average of 65 patients who were PG respondents during that time (Mean=65; SD= 38). Attendings encountered an average of six PG respondent patients with the involvement of a medical student (Mean=6; SD=4).

There were 128 medical students who rotated in the university EDs during the study period (104 fourth-year students, 24 third-year students). Of the 2,753 encounters analyzed, 259 (9.4%) had medical student involvement. Resident physicians were involved in 52% of cases. In the academic ED, 71% of patients had resident involvement in their care and 10% had medical student involvement. In the community ED, 19% of patients had resident involvement in their care and 7.4% had medical student involvement. Characteristics of patients were similar between those with and without medical student involvement (Table 1). Patients with medical student involvement were less likely to also have resident involvement (32% versus 54%). Length of stay was longer for patients with medical students.

**Figure 2:**
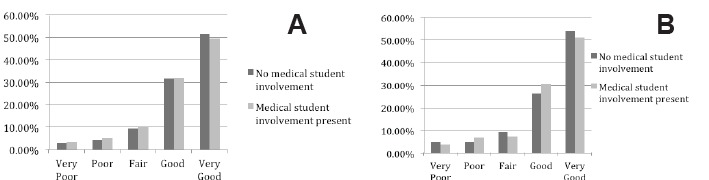
Patient response to primary outcome questions described graphically as a percentage of responses. A, Overall rating of care received during your visit. B, Likelihood of your recommending our emergency department to others.

**Figure 3:**
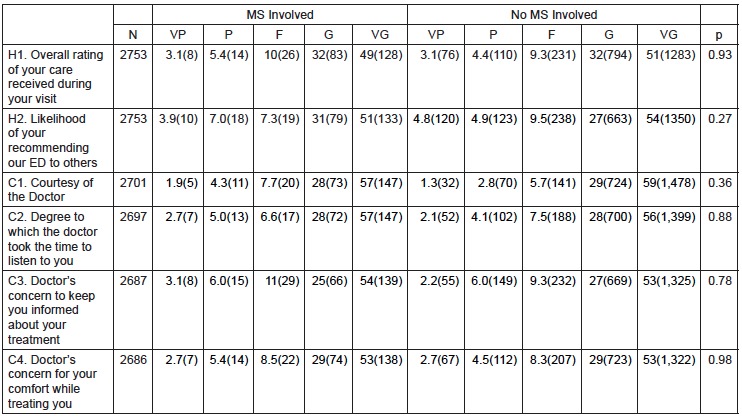
Percentage and number (in parentheses) of patient responses to Press Ganey survey questions about their care by an emergency department stratified by medical student involvement in their care. *MS*, medical student; *N*, number of patient respondents; *VP*, very poor; *P*, poor; *F*, fair; *G*, good; *VG*, very good; *ED*, emergency department

The distribution of responses was similar between encounters with and without medical student involvement for all satisfaction questions. The chi-square tests resulted in p values greater than 0.05 for all PG questions comparing medical student participation to non-medical student participation (see Figure 2 and Figure 3). Most ratings (>80%) were good or very good for each question.

**Figure 4:**
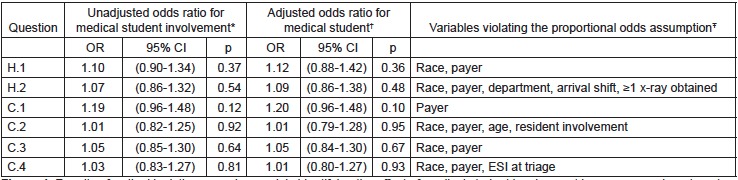
Results of ordinal logistic regression models identifying the effect of medical student involvement in emergency department care in causing decreased patient satisfaction scores. *OR*, odds ratio; *CI,* confidence interval; *ESI*, emergency severity index ^*^Using a univariate ordinal logistic regression model clustered by attending. ^†^Using a partial proportional odds ordinal logistic regression model clustered by attending physician and controlling for: medical student involvement, age, race, department, resident involvement, ESI at triage, primary payer, arrival shift, season of visit, length of stay, ≥1 x-ray obtained, ≥1 computed tomography obtained, and ≥1 laboratory study obtained. ^Ŧ^Violations accounted for in the partial proportional odds models.

In the ordinal regression models, medical student involvement was not significantly associated with PG score for any outcome measure, either in the adjusted or unadjusted analyses. Figure 4 notes which variables violated the proportional odds assumption in each model. There were no significant interactions in any model. Fit was equivalent between models constructed for each question with no appreciable differences in AIC between the models (data not shown). The CIs for medical student involvement were not appreciably wider in the adjusted versus the unadjusted model, indicating that there was likely no instability caused by the number of independent variables in the models.

In the sensitivity analysis, the effect of medical student involvement remained non-significant for all questions when analyzed at each ED site. For question H1 (overall rating of ED care), the results OR for medical student involvement at the academic ED was 1.08 (95% CI [0.81–1.46]) (p=0.587) and for the community ED was 1.11 (95% CI [0.66–1.87]) (p=0.689). For question H2 (likelihood of recommending), the results OR for medical student involvement at the academic ED was 1.02 (95% CI [0.74–1.40]) (p=0.892) and for the community ED was 1.20 (95% CI [0.77–1.87]) (p=0.413).

Table 2 shows descriptive values for the study population compared to the entire discharged ED population. Patients seen in the academic ED, were more likely to be White, with lower ESIs, shorter lengths of stay, and more likely to have private insurance. Other characteristics were similar between EDs, particularly, rates of medical student and resident involvement.

## DISCUSSION

This investigation provides evidence that medical student involvement in emergency care does not adversely impact PG scores. Neither overall ED scores nor the physician provider scores were impacted by medical student involvement. Results were similar across both an academic medical center and an affiliated community teaching hospital.

To the best of our knowledge, this was the first ED study to consider the impact of medical students on PG measures. This distinction is important as PG surveys are generally considered the benchmark for satisfaction goals and may directly impact an institution’s or physician provider’s reputation and financial reimbursement.^4^

The American College of Emergency Physicians (ACEP) recognizes the value of patient satisfaction surveys and makes recommendation regarding optimal features of survey tools.^22^ PG methodology is compliant with most ACEP recommendations including transparency of process and analysis, consideration for education level survey subjects, administration close to service date, and collection of discrete data points.^16^ The basis of criticism of most surveys, including PG, involves the ACEP recommendations that surveys have a statistically valid sample size, and are free from selection bias.^23^,^24^ Despite this limitation, PG is the most commonly used patient satisfaction survey service, and represents the standard across the healthcare industry.^16^ The broad use of PG surveys and comparability across settings, has prompted similar ED patient satisfaction research.^25^–^28^

Our results should provide reassurance to clerkship directors, medical directors, and hospital administrators that student education does not come at the expense of PG scores. This is particularly important since emergency medicine is being increasingly recognized as an important learning experience for medical students.^10^,^11^ Furthermore, this conclusion extends across two very different ED settings.

While most similar research has resulted in improved patient satisfaction from medical student involvement, our findings were neutral. The design of our study does not explain why we found a neutral result. One potential reason may be that the medical student effect is too small to detect in one full year’s worth of patient data. Another potential reason may be the difference in care setting. Patients have reported apprehension regarding student involvement in intimate exams, in cases that are more emotional, and in more serious situations. These are all common occurrences in a busy ED.^6^–^9^ Another potential confounding variable could be longer lengths of stay associated with medical student involvement. We found that length of stay increased by 25 minutes, a finding consistent with other studies.^29^ Further work is needed to explain the impact of medical student involvement on patient satisfaction.

Engaging medical students in the PG survey could improve PG scores. Providing students with patient satisfaction skills might promote better interpersonal interactions and better patient communications.^1^–^4^ Perhaps coaching medical students to more frequently check on patient’s needs could mitigate the longer stays seen with student involvement.^4^

## LIMITATIONS

Our results represent the experience of one institution with an established history of clinical teaching, one group of physicians, and medical students from one medical school. However, we included an academic and a traditional community ED, each with very different staffing models and patient populations. We hope this supports the generalizability of our findings. Although we accounted for clustering at the attending level we did not explore additional levels of clustering, such as specific attending-medical student dyads.

There are several limitations inherent to the PG phone methodology including limited language options, need for patients to own a phone, and an inability to identify true survey response rates. We compensated by trying to compare the survey population to the population of discharged ED patients as a whole. Based on the characteristics of the two study EDs, we believe that the greater rates of Whites and private insurance in the study population were driven by the greater proportion of visits to the academic ED as compared to the overall discharged ED population. Our community ED’s population is more likely to be Black and self-pay. The results of the sensitivity analysis which was limited to the community ED and which were consistent with our overall results provides reassurance that these differences did not cause biased results. Study patients appeared to have shorter length of stay than the discharged population as a whole. However, it is unclear how this may have impacted our outcomes.

We were able to abstract most relevant variables from our EMR except door-to-doctor time, which may have resulted in a degree of unmeasured confounding. There also may be unaccounted differences between third- and fourth-year students, including interest in the rotation and clinical skill. The impact of these potential differences on PG scores is unclear.

## CONCLUSIONS

We found that medical student involvement in ED care does not adversely impact PG scores. Neither overall scores nor physician scores were impacted by medical student involvement at our institution. Further, the results were similar across both an academic medical center and the community teaching hospital.

## Figures and Tables

**Table 1:** Percentage and number (in parentheses) of 2,753 discharged emergency department (ED) patients with completed Press Ganey survey results for the total population and broken down by medical student vs. no medical student participation separately.

Variable	Total (n=2,753)	Medical student involved (n=259)	No medical student involved (n=2,494)
ED visited			
Academic	64% (1,773)	72% (186)	64% (1,587)
Community	36 (980)	28 (73)	37 (907)
Age (in years)*	38 (26–54)	40 (27–55)	38 (25–54)
Race			
African American or Black	38 (1,057)	36 (93)	39 (964)
White or Hispanic	58 (1,588)	61 (159)	55 (1,375)
Other	3.9 (108)	2.7 (7)	6.2 (155)
Season of visit			
Winter (Jan–Mar)	34 (922)	35 (90)	33 (9,832)
Spring (Apr–June)	22 (598)	12 (31)	23 (567)
Summer (July–Sept)	23 (644)	38 (99)	22 (545)
Fall (Oct–Dec)	21 (589)	15 (39)	22 (550)
Time of arrival			
Day shift (7am–3pm)	44 (1,211)	54 (139)	43 (1,072)
Evening shift (3pm–11pm)	40 (1,107)	37 (96)	41 (1,011)
Night shift (11pm–7am)	16 (435)	9.3 (24)	16 (411)
Emergency severity index			
Level 1 & 2	13 (354)	12 (31)	13 (323)
Level 3	54 (1,490)	57 (148)	54 (1,342)
Level 4 & 5	33 (909)	31 (80)	33 (829)
Payer type			
Managed care/private insurance	35 (975)	34 (88)	36 (887)
Medicaid	27 (731)	29 (76)	26 (655)
Medicare	17 (465)	16 (42)	17 (423)
Other government payer	2.7 (75)	3.5 (9)	2.6 (66)
Self pay	18 (507)	17 (44)	19 (463)
ED length of stay (in minutes)*	227 (142–338)	249 (161–355)	224 (141–335)
X-ray was ordered	32 (887)	30 (77)	32 (810)
CT was ordered	15 (419)	18 (46)	15 (373)
Lab tests were ordered	59 (1,613)	61 (158)	58 (1,455)
Providers			
Medical student involved in care	9.4 (259)	-	-
Resident physician involved in care	52 (1,442)	32 (84)	54 (1,358)

*CT,* computed tomography

* Medians and interquartile ranges (IQR) are provided for Age and ED Length of Stay.

**Table 2:** Percentage of 2,753 discharged emergency department (ED) patients with completed Press Ganey (PG) survey results compared to the entire population of patients discharged from the study EDs.

Variable	Patients with PG surveys (n=2,753)	All patients discharged from study EDs (n=111,180)
ED visited		
Academic	64%	55%
Community	36	45
Age (in years)*	38	42 (28–54)
Race		
African American or Black	38	47
White or Hispanic	58	48
Other	3.9	5.0
Season of visit		
Winter (Jan–Mar)	34	30
Spring (Apr–June)	22	24
Summer (July–Sept)	23	24
Fall (Oct–Dec)	21	23
Time of arrival		
Day shift (7am–3pm)	44	37
Evening shift (3pm–11pm)	40	41
Night shift (11pm–7am)	16	21
Emergency severity index (ESI)†		
Level 1 & 2	13	21
Level 3	54	46
Level 4 & 5	33	30
Payer type		
Managed care/private insurance	35	24
Medicaid	27	30
Medicare	17	19
Other government payer	2.7	2.0
Self pay	18	24
ED length of stay (in minutes)*	227 (142–338)	298 (108–347)
X-ray was ordered	32	38
Computed tomography was ordered	15	18
Lab tests were ordered	59	59
Providers		
Medical student involved in care	9.4	8.6
Resident physician involved in care	52	48

* Medians and interquartile ranges (IQR) are provided for Age and ED Length of Stay.

† ESI measurement not available for 2.2% of the entire ED population.
